# The role of a changing Arctic Ocean and climate for the biogeochemical cycling of dimethyl sulphide and carbon monoxide

**DOI:** 10.1007/s13280-021-01612-z

**Published:** 2021-09-04

**Authors:** Hanna I. Campen, Damian L. Arévalo-Martínez, Yuri Artioli, Ian J. Brown, Vassilis Kitidis, Gennadi Lessin, Andrew P. Rees, Hermann W. Bange

**Affiliations:** 1 Department of Chemical Oceanography, GEOMAR Helmholtz Centre for Ocean Research Kiel, Düsternbrooker Weg 20, 24105 Kiel, Germany; 2 Plymouth Marine Laboratory, Plymouth, PL1 3DH UK

**Keywords:** Arctic Ocean, Climate, Carbon monoxide, Dimethyl sulphide, Ice loss, Trace gases

## Abstract

Dimethyl sulphide (DMS) and carbon monoxide (CO) are climate-relevant trace gases that play key roles in the radiative budget of the Arctic atmosphere. Under global warming, Arctic sea ice retreats at an unprecedented rate, altering light penetration and biological communities, and potentially affect DMS and CO cycling in the Arctic Ocean. This could have socio-economic implications in and beyond the Arctic region. However, little is known about CO production pathways and emissions in this region and the future development of DMS and CO cycling. Here we summarize the current understanding and assess potential future changes of DMS and CO cycling in relation to changes in sea ice coverage, light penetration, bacterial and microalgal communities, pH and physical properties. We suggest that production of DMS and CO might increase with ice melting, increasing light availability and shifting phytoplankton community. Among others, policy measures should facilitate large-scale process studies, coordinated long term observations and modelling efforts to improve our current understanding of the cycling and emissions of DMS and CO in the Arctic Ocean and of global consequences.

## Introduction

### The Arctic Ocean plays a central role in global climate dynamics

The Arctic ice cover substantially contributes to the planetary albedo (Thackeray and Hall 2019). Sea ice plays a key role in global biogeochemical cycles. It is a permeable interface for various exchange processes (Loose et al. 2011), including the sea-air exchange of the climate-relevant gases dimethyl sulphide (DMS) and carbon monoxide (CO); and provides an ecosystem for microbial communities involved in the biogeochemical cycling of these compounds (e.g. Xie et al. 2009; Vancoppenolle et al. 2013; Damm et al. 2016). Ongoing global warming due to man-made greenhouse gas emissions lowered the snow and sea ice coverage thereby decreasing albedo, thus further accelerating warming as part of a process called Arctic amplification (Box et al. 2019). This has cascading effects on atmospheric and biophysical processes in the ocean and on land which drives environmental conditions towards an unprecedented state of the Arctic (Box et al. 2019). As the Arctic is integral to the global (climate) system, any changes to environmental conditions have consequences within and beyond the Arctic region affecting climate, communities and economy (e.g. Cohen et al. 2020), where arising economic costs most probably outweigh potential benefits (Alvarez et al. 2020). DMS and CO are chemically reactive in the atmosphere and therefore have the potential to counterbalance or enhance the ongoing changes, depending on the direction of change in their production and loss terms. It is thus pivotal to understand the interaction between ongoing changes and the biochemical cycling of DMS and CO in the Arctic Ocean.

The rapid sea ice loss and permafrost thawing manifests climate change in the Arctic Ocean. It indicates an overarching transition of the Arctic environment since it initiates the modification of numerous biogeochemical processes with far-reaching consequences.

Sea ice decreases rapidly with the largest loss observed in summer (September): 12.8 ± 2.3% ice cover has been lost per decade relative to the 1981–2010 mean, which is equal to sea ice loss of 83 000 km^2^ year^−1^ (IPCC 2019). Enhanced erosion, increased rainfall and greater riverine inputs due to permafrost thawing will flush more and different terrestrial material into the Arctic Ocean (e.g. Stedmon et al. 2011; Box et al. 2019). First-year ice will dominate over multi-year ice and the number of melting ponds, ice-edges and open-ocean like areas will increase (e.g. Meier et al. 2014; Kwok 2018). Ice melting increases the freshwater inputs, which leads to increasing stratification, possibly limiting nutrient remineralisation depending on the region (Lannuzel et al. 2020), and affecting nutrient and trace metal input (e.g. Hopwood et al. 2018).

Light availability and penetration at the ocean surface will increase due to ice loss and the overall decreasing albedo (Pistone et al. 2014). It stimulates an earlier onset of spring blooms and likely regular autumn blooms due to regionally later freeze-up, potentially increasing primary productivity of ice-algae and pelagic phytoplankton (Ardyna and Arrigo 2020).

That, in turn, has multiple consequences for phytoplankton community structure and production (Ardyna and Arrigo 2020). Because of more open-ocean like areas, phytoplankton and bacterial communities are likely to shift. With shrinking multiyear ice the overwintering habitat of sympagic algae will be lost, which will decrease microalgal diversity favouring pelagic or cryo-pelagic species, such as *Phaeocystis sp.* and flagellates (e.g. Lannuzel et al. 2020). The increase of melt pond coverage might support the development of dense algal colonies, e.g. formed by the under-ice pelagic diatom *Melosira arctica* (Assmy et al. 2017). With regional and seasonal heterogeneity, primary productivity is predicted to generally increase in both sea ice and seawater in the Arctic, being possibly constrained by nutrient availability (Vancoppenolle et al. 2013). However, between 2012 and 2018 chlorophyll a concentration in Arctic Ocean surface waters increased 16 times faster than before, suggesting an increased primary production sustained by an additional input of nutrients due to sea ice melt, mixing at shelf breaks or advection from lower latitudes (Ardyna and Arrigo 2020).

Changes in bacterial communities are also likely and closely linked to seasonal ice melting and changes in primary productivity. Thus, heterotrophic activity is likely to increase as it is mostly driven by primary productivity (Lannuzel et al. 2020). The SAR11 clade (Pelagibacterales) is the most abundant and ubiquitous clade of the bacterial communities worldwide, yet its variation differs among habitats in the Arctic Ocean (Han et al. 2014). In the Chukchi Sea, for example, a few bacterial groups, including species belonging to *Roseobacter* (Malmstrom et al. 2007), dominate community composition and biomass production. For the abundance, production, species composition and activity of under-ice bacterioplankton and bacteria in general, dissolved organic matter (DOM)—often released by pulses of seasonal melting first year ice—is probably a dominant factor (Underwood et al. 2019). Indeed, Jackowski et al. (2020) found that dissolved organic carbon (DOC) was the dominant factor for bacterial production. Moreover, phytoplankton community composition also affects availability and characteristics of DOM and semi-labile dissolved organic carbon, favouring certain bacterial strains (Tisserand et al. 2020). However, DOM and DOC bioavailability decreases strongly from summer to autumn (Jackowski et al. 2020) making it sensitive to climate change-related impacts on Arctic seasonality.

Ocean acidification significantly affects high latitude and Arctic waters. These regions are sensitive to ocean acidification having naturally high concentrations of dissolved inorganic carbon and low alkalinity concentrations, which has far reaching consequences on phytoplankton and bacterial communities (Amundsen et al. 2013; Terhaar et al. 2020)*.* Sea ice melting and permafrost thawing could even enhance ocean acidification by increasing river and glacial runoff and enhancing terrestrial organic carbon loading (Semiletov et al. 2016). Model results suggest that the pH in the surface waters of the Arctic Ocean could decrease by about 0.45 by the end of this century (Terhaar et al. 2020), although the decrease in pH might show a high regional heterogeneity.

Gas fluxes between ocean and atmosphere may be altered and enhanced by sea ice loss. In particular, less ice implies a stronger transfer of energy from wind to the ocean, with more waves, turbulence, mixing, and increased sea ice mobility, which, in turn, will enhance the air-sea gas exchange (e.g. Meneghello et al. 2018).

### DMS and CO play important roles in atmospheric chemistry and climate

**Fig. 1:**
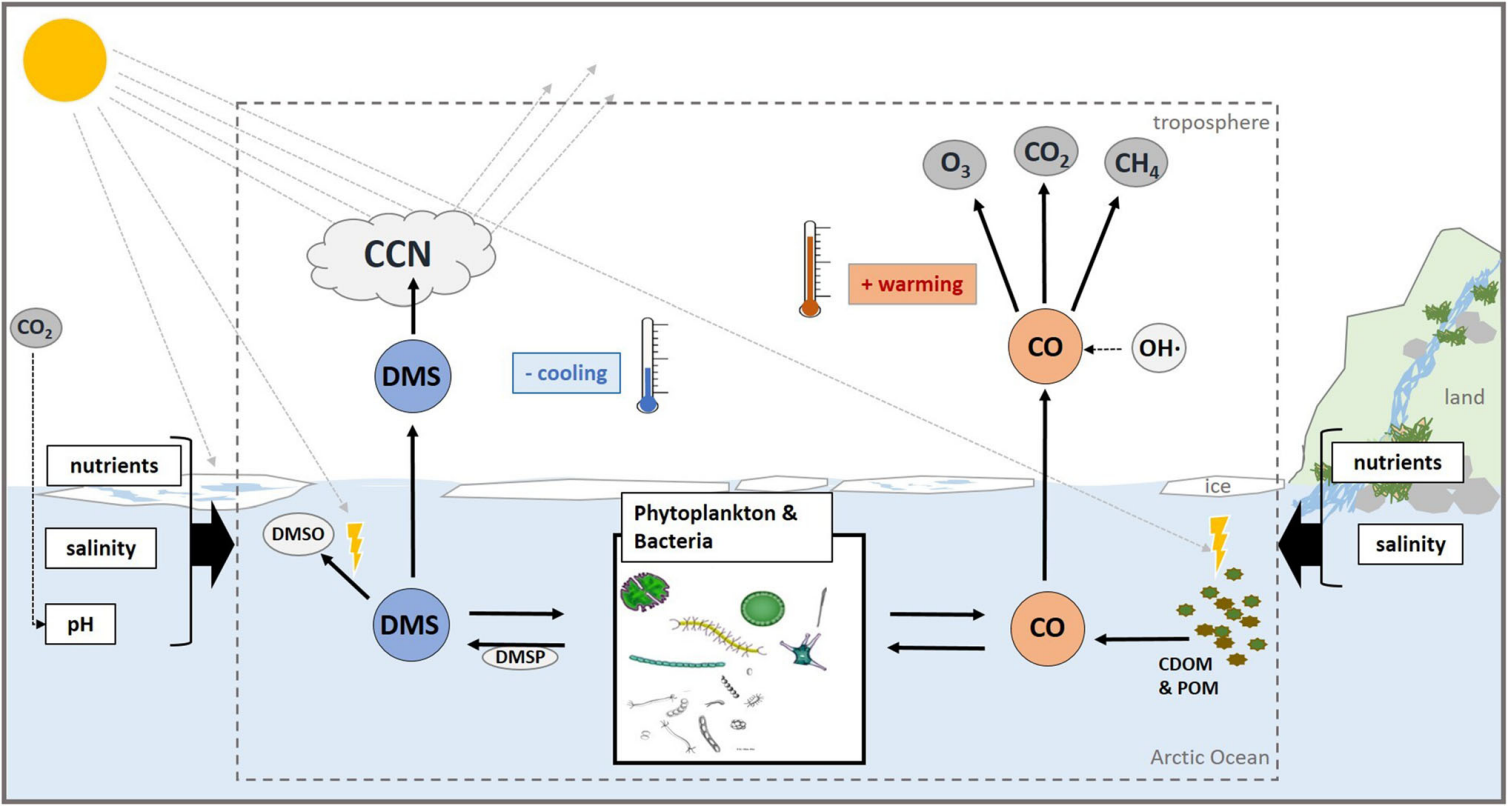
Schematic view of the interactions of DMS and CO production, consumption and emission pathways in a changing Arctic Ocean. The dashed box marks the processes discussed in this article, comprising interactions in the ocean, ice and atmosphere. Thick arrows outside and towards the box represent changes in nutrients, salinity or pH due to increased ice melt (left side) and/or increased material input from land (right side). Those potentially alter ice-associated and pelagic DMS and CO processes and thus emissions in an uncertain way

Figure 1 shows schematically their key processes and fluxes in the Arctic Ocean. DMS has the potential to counteract warming by increasing the regional albedo**.** DMS, as the precursor of sulphate aerosols, affects the concentration of cloud condensation nuclei (CCN), which in turn increases the formation of clouds, thus the Earth’s albedo, and potentially cools the atmosphere (e.g. Charlson et al. 1987; Korhonen et al. 2008; Park et al. 2021). This DMS-driven ocean–atmosphere interaction could counteract the decreasing albedo in the Arctic, which could be particularly important in summer when the aerosol burden is low (Mungall et al. 2016).

Furthermore, DMS plays a role in the oxidation pathways of climate-relevant gases. These include isoprene, ammonia and organohalogens (Hopkins et al. 2020), as well as the potent greenhouse gas methane which, like DMS, can be microbially produced from di-methyl-sulphonio-propionate (DMSP) depending on environmental conditions in the Arctic Ocean (Damm et al. 2015).

DMS occurs globally in association with phytoplankton in surface waters, whereby biologically productive waters around the Arctic pack ice represent a strong DMS source (Levasseur 2013). DMS is produced by the enzymatic DMSP breakdown by heterotrophic bacteria and as a metabolic product in algae (both, planktonic microalgae and macroalgae) (e.g. Stefels 2000). In algae, DMSP may be involved in various cellular processes, such as regulation of the algal carbon and sulphur metabolism via an overflow mechanism and fulfilling physiological functions including osmoregulation, cryo-protection, and protection against oxidative stress (e.g. Stefels 2000; Sunda et al. 2002). Yet, its cellular function is not entirely understood. Intracellular DMSP concentrations can vary strongly between major microalgal groups, and thus the distribution of DMSP and DMS in the ocean depends on microalgae community composition. Dinoflagellates and prymnesiophytes are strong DMS producers, and diatoms are weak DMS producers (Levasseur 2013). DMS production also depends on the physiological status of the algae and environmental stressors such as nutrient limitation and ultraviolet light (Sunda et al. 2002). If DMSP is released into the water column, it is by active exudation, autolysis, viral lysis, and grazing by zooplankton (Stefels et al. 2007). Pelagic bacteria generally either cleave it, generating DMS, or metabolise it to other sulphur compounds by demethylating/demethiolating DMSP to methyl-mercaptopropionate, methanethiol or inorganic sulphur (Stefels 2000). Other major loss processes for oceanic DMS are photochemical oxidation to dimethylsulphoxide (DMSO) and release to the atmosphere via air-sea gas exchange (Levasseur 2013). Given the tight connection between the cycling of marine DMS and microalgae, changes in the Arctic Ocean phytoplankton community structure, further warming and decrease in sea ice coverage can therefore lead to changes in the production and emission of DMS.

Oceanic DMS emissions amount to 17–34 Tg sulphur (S) year^−1^, which represents 80–90% of all marine biogenic S emissions, and up to 50% of global biogenic S emissions (Levasseur 2013). Yet, only ~ 10% of the DMS produced by plankton finds its way to the atmosphere (Bates et al. 1994) because the majority of dissolved DMS is oxidized microbially and photochemically in seawater (e.g. Levasseur 2013). In the Arctic region, oceanic DMS emissions could cause a significant cooling effect following enhanced CCN formation (Mungall et al. 2016; Hopkins et al. 2020; Park et al. 2021; Qu et al. 2021).

CO is an indirect greenhouse gas with a radiative forcing nearly twice that of carbon dioxide (CO_2_) on a molecular basis (IPCC 2013). Its presence in the atmosphere triggers a series of reactions increasing other greenhouse gases such as CO_2_, methane and ozone (O_3_): it reacts with hydroxyl radicals (OH·) to form CO_2_ and it outcompetes methane in the reaction with tropospheric OH·, prolonging its atmospheric lifetime (Conte et al. 2019). Moreover, CO affects the ozone concentrations in the troposphere, where O_3_ acts as a strong greenhouse gas (Dignon and Hameed 1985). The lifetime of tropospheric CO is ~ 2 months (Prather 1996).

Both biological and abiotic processes produce CO in the surface ocean, whereas microbial uptake, mixing to subsurface layers and exchange to the atmosphere represent its loss terms (Conte et al. 2019). CO is produced photochemically through the reaction of ultraviolet (UV) or blue light with either coloured dissolved organic matter (CDOM) (Zafiriou et al. 2008) or, to a lesser extent, particulate organic matter (POM) (Xie et al. 2009), e.g. from ice algae (Song and Xie 2017). Dark (thermal) production and biological production by phytoplankton are additional small sources (Zhang et al. 2008; Tran et al. 2013). Microbial uptake is the major sink of CO in marine waters (Conte et al. 2019), but details of its physiological function and kinetics are still uncertain. Release to the atmosphere via air-sea gas exchange represents a minor loss term (Conte et al. 2019). The photochemical and microbial-driven sources and sinks of CO could be altered by warming, increased light penetration because of sea-ice loss and increase of DOM inputs to the Arctic Ocean.

The ocean’s surface is ubiquitously supersaturated with CO (Conte et al. 2019). Yet air-sea gas exchange is the smallest contributor to the atmospheric budget of CO contributing only ~ 1% of the natural atmospheric source. However, there are large uncertainties in the magnitude of the global marine CO emissions: Recent estimates range between ~ 9 Tg CO year^−1^ and 20 Tg CO year^−1^ (Conte et al. 2019; Zheng et al. 2019). To date there are no regional CO emission estimates from the Arctic Ocean. In the Arctic Ocean, CO measurements are scarce, especially within sea ice and at the sea surface microlayer. The few available studies on CO in the Arctic Ocean report elevated and highly variable concentrations compared to other ocean basins (Tran et al. 2013). Studies by Xie et al. (2005) and Song et al. (2011) found high CO concentrations in bottom sea ice suggesting a link between CO production and ice algae blooms.

Changes of the biogeochemical processes described previously might have substantial consequences on Arctic Ocean DMS and CO concentrations and fluxes. The production and consumption pathways of DMS and CO depend on light, microalgal and bacterial community structure as well as on CDOM and POM. DMS and CO air-sea gas exchange is regulated by the presence of sea-ice, stratification, wind speed, temperature and salinity. All these processes are directly or indirectly affected by the ongoing environmental changes in the Arctic Ocean such as the loss of sea ice. To this end, we assess here the potential consequences of the ongoing environmental changes for future DMS and CO biogeochemical pathways and emissions in the Arctic Ocean, identify key knowledge gaps and point to potential future research needs that should be supported by international policy frameworks.

### Potential impacts of ongoing environmental changes on DMS and CO cycling in the Arctic Ocean

In the following section, we discuss the ongoing changes that might play a role for DMS and CO production and consumption processes to be addressed by future studies.

#### Direct impacts of ice melting

The melting of ice on land and at sea, in addition to permafrost thawing will affect the Arctic Ocean’s ecosystems and biogeochemical processes with potential consequences for DMS and CO cycling.

Highly productive ice algae responsible for high DMS concentrations are known to inhabit Arctic sea ice (Levasseur 2013). Thus, the loss of sea ice could lead to a decrease in DMS/P production, or a change in the ratio of ice-associated and open ocean DMS producers promoting the latter (Lannuzel et al. 2020). However, in the phase of increased melting, ice edge effects may stimulate DMS/P production as indicated by elevated DMS/P values in partially ice-covered regions (Jarníková et al. 2018). The results show that DMS production is inextricably linked with the prevailing phytoplankton and microbial communities in both ice-associated and pelagic habitats. Since the ongoing changes strongly affect phytoplankton and microbial communities, changes therein have to be fully considered for a better understanding of future DMS dynamics.

Enhanced ice melting could increase CO production in Arctic surface waters. Increasing regions of ice-melt may lead to higher CDOM and POM supply, which—when coupled to greater light availability—may increase photochemical CO production (Song et al. 2011). Also progressively thinner sea ice could lead to increased light penetration and CO production within the ice bottom layer. Since ice then will be permeable more frequently (Vancoppenolle et al. 2013) it increases the amount of CO that can be released to the atmosphere (Song et al. 2011). Additionally, melting and refreezing of seawater, which could happen more frequently in the future, may lead to higher CO concentrations (Xie and Gosselin 2005), being in line with elevated CO concentrations coinciding with higher CDOM absorbance and more intense stratification due to ice melting (Tran et al. 2013).

Enhanced permafrost thawing and erosion could additionally increase CO photoproduction in the coastal regions of the Arctic Ocean. Especially in the Eurasian basin, it could increase the availability of CDOM and POM via increased riverine input, and potentially alter their spectral characteristics. For instance, in winter, older and more refractory organic material would be exported (Stedmon et al. 2011). Moreover, enhanced erosion of soils potentially increases terrestrial CDOM and POM loads in coastal waters, which could lead to higher CO photoproduction compared to its photoproduction via marine CDOM (Song and Xie 2017).

#### Increasing light availability

Ice melting increases the light availability in the Arctic Ocean, possibly resulting in both an increase in biological DMS production (Levasseur 2013) and in CO photoproduction (Song et al. 2011). Whether this will also increase emissions, depends however on further production and consumption terms and their reply to ongoing changes.

Increasing light availability in the Arctic Ocean promotes higher primary productivity due to changes in seasonality. An earlier onset of the bloom increasing the phytoplankton and ice algae biomass could enhance the overall biological production of DMS/P (Lannuzel et al. 2020). However, the fraction of DMSP eventually resulting in DMS emissions depends on the abundance and taxonomy of microalgae, bacterial activity and further environmental conditions (Levasseur 2013), which all underlie large regional variations. Since UVA light is responsible for 60–75% of the DMS photooxidation in the sunlit surface (Taalba et al. 2013), increasing UV-A light could increase the photooxidation of DMS to DMSO, thereby decreasing DMS surface concentrations.

As more light becomes available and penetrates the surface Arctic Ocean when the sea ice retreats, it possibly increases both photochemical and biological CO production over the year. For CO photoproduction, Song and Xie 2017 show that POM dominates over CDOM as a source in the bottom of sea ice. POM content could increase even more in the future as primary productivity increases (e.g. Lannuzel et al. 2020), which could promote both photochemical and biological CO production by phytoplankton. However, that depends also on the community composition and possible shifts therein.

#### Changing phytoplankton community

Increasing light and open-ocean conditions will probably lead to increased phytoplankton growth and shift towards cryo-pelagic and pelagic species. Shifts in the phytoplankton community will likely influence DMS and CO concentrations in surface Arctic waters, potentially affecting their emissions to the atmosphere.

Expected phytoplankton community shifts will likely lead to an overall increase in DMS production. Its precursor molecule, DMSP, largely depends on the plankton community composition, in particular on the abundance of strong DMSP producers with DMSP-lyase activity (Levasseur 2013). Strong DMSP producers are Dinoflagellates and primnesiophytes such as *Phaeocystis* and *E. huxleyi*, which may increase in abundance in future (Assmy et al. 2017). Due to their high intracellular concentrations of DMSP it was suggested that their biomass governs DMSP production (Stefels 2000), also in the Arctic Ocean (Park et al. 2018). Weaker DMSP producing species, however, may contribute significantly to DMSP production when being under stress, e.g. nutrient limitation or high UV light (Levasseur 2013). Both potentially increase in future (Vancoppenolle et al. 2013).

Expected changes in the phytoplankton community distribution could point to an increase in microalgal CO production. During ice algal blooms, large CO accumulations were observed in the lowermost sea ice layer (Song et al. 2011), which they may produce directly or indirectly, via CDOM input. Laboratory experiments indicated that cyanobacteria and diatoms are large CO emitters (Gros et al. 2009). However, only one field study so far has confirmed biological CO production and observed *Phaeocystis *sp., dinoflagellates and to lesser extent diatoms to produce CO in the Arctic Ocean (Tran et al. 2013). Given that *Phaeocystis *sp., flagellates and several diatom species abundances will probably increase in the future (Lannuzel et al. 2020), this could enhance CO production in the Arctic Ocean. Hence, although biological CO production in the Arctic Ocean is of minor importance today, it might become more pronounced in the future.

#### Changing bacterial community

The microbial community may be profoundly altered by changes in seasonality of organic matter supply and algal community structure, which could greatly affect DMS cycling and both CO production and consumption in the water column. Especially changes in the *Roseobacter* clade may be important, given its role in the biogeochemical cycle of both gases.

The composition of the prokaryotic community is as important for DMS production as the phytoplankton community composition (Levasseur 2013). The abundant marine α-proteobacteria *Roseobacter* catabolize DMSP in high amounts by several mechanisms (Todd et al. 2012), which field studies confirmed for an Arctic fjord (Kongsfjorden) (Zeng et al. 2016). Sipler et al. (2017) found that some taxa within the *Roseobacter* clade would thrive in a changing Arctic Ocean. Arctic field studies indicate that bacteria use DMSP mostly as a carbon source, with conversion efficiencies of DMSP into DMS of up to 30% (Motard‐Côté et al. 2012). DMS seems to be used as an auxiliary S source by the same clades that consume DMSP (Levasseur 2013). Thus, bioavailability and characteristics of DOM, which determine bacterial community compositions (Jackowski et al. 2020), potentially also govern DMSP to DMS conversion, and thereby, whether DMS is released to the atmosphere.

Changes in the bacterial community could also alter CO concentration in surface waters and ice, with microbial uptake being the major CO sink (Xie et al. 2009). Diverse communities of marine bacteria are oxidizing CO at environmentally relevant rates (King and Weber 2007) with the marine *Roseobacter* group being among those with highest specific rates. However, large uncertainties about dominant CO oxidizers remain (King and Weber 2007). CO oxidation capability is indicated by holding both forms of the gene *coxL* (Cunliffe 2011). *Roseobacter* uses CO mainly as a supplemental energy source, next to DOM (Moran and Miller 2007). Thus, CO oxidation could help heterotrophic bacteria to survive carbon limitation in changeable environments (Cordero et al. 2019), such as the Arctic. Yet, the physiological details remain unclear. This emphasizes that future research should focus on the relationship between bacterial species composition, their CO oxidation capability and rates, and the resulting CO concentrations as also suggested by Tran et al. (2013). Studies of the microbial CO consumption rates propose that in Arctic waters (Beaufort Sea) the CO microbial consumption depends indeed on bacterial activity which, in turn, mainly follows primary productivity (Xie et al. 2005). This may suggest an increase in CO consumption, which would mean a decrease in CO concentrations in the surface layer and thus in CO emissions. Comparing CO consumption rates in spring and autumn in the Beaufort Sea indicated that bacterial community shifts largely dominate the CO consumption, resulting in much lower rates, followed by higher CO emissions, in spring than in autumn (Xie et al. 2009). This phenomenon might be intensified in future due to an earlier onset of primary productivity and more ice edges and melt ponds in the Arctic Ocean (Xie and Gosselin 2005).

#### Ocean acidification

Ocean acidification is taking place rapidly in polar oceans with consequences especially for DMS and probably also for CO, since their production is linked to phytoplankton and bacterially mediated processes.

Studies of DMS production under ocean acidification in the Arctic Ocean reveal contradictory results. Hopkins et al. (2020) provide a comprehensive overview of studies on DMS production under ocean acidification. It shows that future DMS production rates in the Arctic Ocean with lower pH do not show a general increasing or decreasing trend and depend on season, location and experimental approach. Two mesocosm studies showed a decrease of DMS due to decreasing DMSP to DMS conversion by bacteria (Archer et al. 2013; Hussherr et al. 2017). However, several microcosm studies indicated DMS production and emissions to be resilient to ocean acidification in the Arctic (Hopkins et al. 2020). This is in line with the ability of polar bacteria to cope with strong pH fluctuations over the range 7.5–8.3 (Hoppe et al. 2018) emphasizing the close link between DMS concentrations and bacterial activity and metabolism under ocean acidification.

It is uncertain whether ocean acidification affects CO production. Gao and Zepp (1998) showed that photochemical breakdown of CDOM increased with very low pH (5.5). This could be a hint that ocean acidification may enhance the photochemical formation of CO, particularly in the Arctic Ocean because of increased riverine CDOM inputs (see above). However, this idea needs to be investigated with pH levels characteristic for the Arctic Ocean. Further, it may alter CO production indirectly via the influence on bacterial and phytoplankton processes affecting also the CDOM and POM pool (Hopkins et al. 2018).

#### Physical properties

The melting of sea ice could alter energy transfer between the ocean and the atmosphere, and result in an increased air-sea gas exchange. Although this is likely to happen with high regional variations, it will strongly affect DMS and CO fluxes between the ocean and atmosphere.

Rising temperatures of surface waters and sea ice melting could increase concentrations and sea-air fluxes of DMS (Bock et al. 2021). In the open Arctic Ocean, DMS gradients seem to co-occur with strong surface temperature and salinity gradients, suggesting that oceanographic fronts could play a role for changes in DMS concentration (Jarníková et al. 2018), and which could occur more often in future with sea ice melting. The annual DMS flux from the Arctic Ocean to the atmosphere was estimated to increase by more than 80% by 2080, and could significantly change summer aerosol concentrations and the radiative balance in the Arctic region (Gabric et al. 2005). However, it is not yet clear to what extent increasing DMS emissions will add to the atmospheric DMS mole fractions (Levasseur 2013). Models that incorporate sea ice DMS production into DMS emission estimates, show that first year ice enhances DMS production by 18% and DMS release to the atmosphere by 20–26% (Hayashida et al. 2017). This indicates that under-ice DMS production contributes significantly to DMS emissions in the Arctic Ocean when the ice is melting (Elliott et al. 2012) and therefore ice loss might reduce DMS emissions. However, less snow accumulation could promote DMS release to the atmosphere, potentially further enhanced by increasing sea ice mobility, whereas increasing rain would promote DMS deposition to the water column (Lannuzel et al. 2020).

Increasing stratification could increase future CO emissions from the Arctic Ocean. Higher CO concentrations coincided with intensified stratification (e.g. in the Greenland Sea) compared to other regions (Tran et al. 2013). Moreover, CO surface concentrations within the upper 10 m of the water column were significantly higher when the mixed-layer depth was reduced in combination with an increase of CDOM (Tran et al. 2013). These observations may speak for increasing CO emissions in future, since they can be explained by physical properties such as temperature, salinity, water movement and wind, which are all influenced by the retreat and higher mobility of sea ice (Vancoppenolle et al. 2013; Lannuzel et al. 2020). However, most important for the overall CO emissions probably is microbial CO consumption, because it determines the time CO resides in the surface (Tran et al. 2013).

Table 1 summarizes the expected changes discussed above, and their potential impact on DMS and CO Arctic surface water concentration and their emissions from the Arctic Ocean.

**Table 1:** Summary of the expected changes discussed in this article, and their potential impact on DMS and CO Arctic surface water concentration and their emissions from the Arctic Ocean

	DMS	CO
Ice melting	±	+
Increasing light availability	+	+
Changes in phytoplankton community	+	+
Changes in bacterial community	?	?
Ocean acidification	?	?
Air-sea gas exchange	+	±

*+* probable increase, *±* might be balanced, *?* uncertain

## Knowledge gaps and possible directions of future research

To start unravelling the relationship between bacterial activity and community structure and DMS and CO dynamics, it is crucial to understand the bacterial species distribution and its impact on DMS production and microbial CO consumption. Molecular analysis of specific gene abundances from seawater samples combined with in situ trace gas measurements will help to answer open questions: What do microbial CO consumption rates depend on? Which bacterial strains are mainly responsible for DMSP to DMS conversion and CO consumption, and what does their activity depend on? Since the *Roseobacter* clade is known to metabolise both DMSP and CO (Cunliffe 2011), changes concerning that clade might be worth investigating further. However, there are other bacterial groups, like non-*Roseobacter* alphaproteobacteria and gammaproteobacteria (King and Weber 2007; Levasseur 2013) metabolising one or both gases, and which will have implications, too. Hence, dominant bacterial strains involved in DMS or CO cycling should be identified and further investigated.

The processes involved in DMS cycling will likely change under the ongoing changes of the Arctic environment. For an improved understanding of the impacts of ocean acidification on DMS cycling it is crucial to further unravel the physiological (inner cell) role of DMSP. Being a pivotal step within DMS formation, the transformation of DMSP to DMS (Archer et al. 2013) should be incorporated into future experimental design. Those should consider the role of communities of phytoplankton, grazers and bacteria, the impact of their spatial and seasonal variability as well as potential shifts due to sea ice loss (Hopkins et al. 2020).

There are only two studies dealing with CO dynamics and sea ice (Song et al. 2011; Song and Xie 2017). Thus, further detailed investigations of CO processes within and in vicinity to sea ice as well as in melting areas are of importance when trying to predict future developments. Impacts of CDOM and POM spectral characteristics related to their origin (e.g. terrestrial vs. marine) on CO photoproduction also should be addressed, since those likely change with increased material inflow (Fig. 1). Since biological CO production in the Arctic Ocean may become more pronounced, further Arctic field studies, e.g. mesocosm and ice-related studies, are needed to identify dominant CO producers. Standardization and quality-control of CO measurements is a necessary step forward, since to date we have no internationally accepted quality standard for CO in seawater as it is the case for CO_2_, nitrous oxide and methane (Krahmann et al. 2021). The only available reference is the quality-control threshold for atmospheric CO to which several groups try to abide by measuring reference gases calibrated against either NOAA (National Oceanic and Atmospheric Administration) or WMO (World Meteorological Organization) standard scales (Krahmann et al. 2021). In light of the ongoing environmental changes, it is pivotal to understand them in detail to improve model parameterizations of fluxes and emission of DMS and CO for future projections. For high latitudes, models underestimate the simulated CO concentrations compared to in situ measurements, perhaps because they lack the CDOM supply by sea ice and rivers, and their biological consumption term seems not suitable (Conte et al. 2019). Process based models (e.g. Kwon et al. 2020) are useful tools to understand drivers of spatial and temporal variability of CO dynamics and to project the integrated effect of all the changes on CO production.

## Societal and policy implications

A resilient and sustainable Arctic environment contributes to global human prosperity. Sustaining its present and future functioning should thus be a main priority of political and societal planning activities. Anthropogenic global warming initiated an overarching transition of the Arctic region, which manifested in the rapid retreat of sea-ice and the thawing of permafrost, which subsequently are affecting numerous natural processes at regional and global level. In this paper, we have discussed how ongoing changes in the Arctic Ocean such as for instance warming and acidification might alter the biogeochemical cycling of the climate-relevant gases DMS and CO (see Table 1). The production of both gases is likely to increase in the future and yet the direction and magnitude of the emissions to the atmosphere as well as the expected long-term feedbacks on regional climate remain uncertain. Hence, despite of their major role in climate, elucidating the socio-economic influence of long-term changes in the production and emissions of DMS and CO will need extensive multidisciplinary efforts in basic research, as well as adequate transfer of knowledge to stakeholders and policy makers. To this end, we propose to:

1. Increase understanding
  - Support international, multidisciplinary studies as well as sustained observations of DMS and CO in the AO. Knowledge transfer between in situ observations and global coupled models should then help establishing more reliable emission projections for DMS and CO, and their effects on the Arctic Ocean.
  - Facilitate research co-design by integrating economics, natural and social sciences, and stakeholders to raise awareness across disciplines of the importance and environmental feedbacks caused by the alterations in DMS and CO emissions in the context of ongoing climate change. This approach could provide societal-relevant knowledge and solutions to holistically manage the implications of Arctic change (Alvarez et al. 2020).
2. Think economy long term
  - Stick to the Paris agreement and decrease global greenhouse gas emissions because this would reduce warming and ocean acidification as these changes could directly and indirectly affect DMS and CO cycling in the Arctic Ocean (see Table 1).
  - Burning of both fossil fuels and biomass (e.g. forest fires) are the major sources of atmospheric CO. To this end CO emissions from these sources must be avoided, e.g. by (i) regulating offshore industrial activities including Arctic ship traffic and oil/gas drilling and (ii) minimizing forest fires in the countries around the Arctic Ocean.
3. Increase general knowledge and acceptance of sustainable political measures
  - Support outreach activities aiming to inform the general public on the key role of atmospheric trace gases for the Arctic Ocean and global climate and the complexity and interconnectedness of natural processes.
  - Promote a societal mind-set shift towards the realization of us being a part of nature, meaning that human wellbeing relies on suitable environmental conditions, to increase the acceptance of sustainable political and economic decisions (see Ives et al. 2018).

## References

[CR1] Alvarez J, Yumashev D, Whiteman G (2020). A framework for assessing the economic impacts of Arctic change. Ambio.

[CR2] Amundsen, H., L. Anderson, A. Andersson, K. Azetsu-Scott, R. Bellerby, M. Beman, H. I. Browman, C. Carlson, et al. 2013. AMAP assessment 2013: Arctic Ocean acidification.

[CR3] Archer SD, Kimmance SA, Stephens JA, Hopkins FE, Bellerby RGJ, Schulz KG, Piontek J, Engel A (2013). Contrasting responses of DMS and DMSP to ocean acidification in Arctic waters. Biogeosciences.

[CR4] Ardyna M, Arrigo KR (2020). Phytoplankton dynamics in a changing Arctic Ocean. Nature Climate Change.

[CR5] Assmy P, Fernández-Méndez M, Duarte P, Meyer A, Randelhoff A, Mundy CJ, Olsen LM, Kauko HM (2017). Leads in Arctic pack ice enable early phytoplankton blooms below snow-covered sea ice. Scientific Reports.

[CR6] Bates TS, Kiene RP, Wolfe GV, Matrai PA, Chavez FP, Buck KR, Blomquist BW, Cuhel RL (1994). The cycling of sulfur in surface seawater of the northeast Pacific. Journal of Geophysical Research.

[CR7] Bock J, Michou M, Nabat P, Abe M, Mulcahy JP, Olivié DJL, Schwinger J, Suntharalingam P (2021). Evaluation of ocean dimethylsulfide concentration and emission in CMIP6 models. Biogeosciences.

[CR8] Box JE, Colgan WT, Christensen TR, Schmidt NM, Lund M, Parmentier F-JW, Brown R, Bhatt US (2019). Key indicators of Arctic climate change: 1971–2017. Environmental Research Letters.

[CR9] Charlson RJ, Lovelock JE, Andreae MO, Warren SG (1987). Oceanic phytoplankton, atmospheric sulphur, cloud albedo and climate. Nature.

[CR10] Cohen J, Zhang X, Francis J, Jung T, Kwok R, Overland J, Ballinger TJ, Bhatt US (2020). Divergent consensuses on Arctic amplification influence on midlatitude severe winter weather. Nature Climate Change.

[CR11] Conte L, Szopa S, Séférian R, Bopp L (2019). The oceanic cycle of carbon monoxide and its emissions to the atmosphere. Biogeosciences.

[CR12] Cordero PR, Bayly K, Leung PM, Huang C, Islam ZF, Schittenhelm RB, King GM, Greening C (2019). Atmospheric carbon monoxide oxidation is a widespread mechanism supporting microbial survival. The ISME Journal.

[CR13] Cunliffe M (2011). Correlating carbon monoxide oxidation with cox genes in the abundant marine Roseobacter clade. The ISME Journal.

[CR14] Damm E, Thoms S, Beszczynska-Möller A, Nöthig E-M, Kattner G (2015). Methane excess production in oxygen-rich polar water and a model of cellular conditions for this paradox. Polar Science.

[CR15] Damm E, Nomura D, Martin A, Dieckmann GS, Meiners KM (2016). DMSP and DMS cycling within Antarctic sea ice during the winter–spring transition. Deep Sea Research Part II.

[CR16] Dignon J, Hameed S (1985). A model investigation of the impact of increases in anthropogenic NOxemissions between 1967 and 1980 on tropospheric ozone. Journal of Atmospheric Chemistry.

[CR17] Elliott S, Deal C, Humphries G, Hunke E, Jeffery N, Jin M, Levasseur M, Stefels J (2012). Pan-Arctic simulation of coupled nutrient-sulfur cycling due to sea ice biology: Preliminary results. Journal of Geophysical Research: Biogeosciences.

[CR18] Gabric AJ, Qu B, Matrai P, Hirst AC (2005). The simulated response of dimethylsulfide production in the Arctic Ocean to global warming. Tellus B.

[CR19] Gao H, Zepp RG (1998). Factors influencing photoreactions of dissolved organic matter in a coastal river of the Southeastern United States. Environmental Science & Technology.

[CR20] Gros V, Peeken I, Bluhm K, Zöllner E, Sarda-Esteve R, Bonsang B (2009). Carbon monoxide emissions by phytoplankton: Evidence from laboratory experiments. Environmental Chemistry.

[CR21] Han D, Kang I, Ha HK, Kim HC, Kim O-S, Lee BY, Cho J-C, Hur H-G (2014). Bacterial communities of surface mixed layer in the Pacific sector of the western Arctic Ocean during sea-ice melting. PLoS ONE.

[CR22] Hayashida H, Steiner N, Monahan A, Galindo V, Lizotte M, Levasseur M (2017). Implications of sea-ice biogeochemistry for oceanic production and emissions of dimethyl sulfide in the Arctic. Biogeosciences.

[CR23] Hopkins FE, Nightingale PD, Stephens JA, Moore CM, Richier S, Cripps GL, Archer SD (2018). Dimethylsulfide (DMS) production in polar oceans may be resilient to ocean acidification. Biogeosciences Discuss.

[CR24] Hopkins FE, Suntharalingam P, Gehlen M, Andrews O, Archer SD, Bopp L, Buitenhuis E, Dadou I (2020). The impacts of ocean acidification on marine trace gases and the implications for atmospheric chemistry and climate. Proceedings of the Royal Society A.

[CR25] Hoppe CJM, Wolf KK, Schuback N, Tortell PD, Rost B (2018). Compensation of ocean acidification effects in Arctic phytoplankton assemblages. Nature Climate Change.

[CR26] Hopwood MJ, Carroll D, Browning T, Meire L, Mortensen J, Krisch S, Achterberg EP (2018). Non-linear response of summertime marine productivity to increased meltwater discharge around Greenland. Nature Communications.

[CR27] Hussherr R, Levasseur M, Lizotte M, Tremblay J-É, Mol J, Thomas H, Gosselin M, Starr M (2017). Impact of ocean acidification on Arctic phytoplankton blooms and dimethyl sulfide concentration under simulated ice-free and under-ice conditions. Biogeosciences.

[CR28] IPCC. 2013. IPCC Climate Change: The Physical Science Basis. In *Contribution of Working Group I to the Fifth Assessment Report of the Intergovernmental Panel on Climate Change*, ed. T.F. Stocker, D. Qin, G.-K. Plattner, M. Tignor, S.K. Allen, J. Boschung, A. Nauels, Y. Xia, et al. Cambridge University Press, Cambridge, UK and New York, NY, USA

[CR29] IPCC. 2019. Meredith, M., M. Sommerkorn, S. Cassotta, C. Derksen, A. Ekaykin, A. Hollowed, G. Kofinas, A. Mackintosh, J. Melbourne-Thomas, M.M.C. Muelbert, G. Ottersen, H. Pritchard, and E.A.G. Schuur. Polar Regions. In *IPCC Special Report on the Ocean and Cryosphere in a Changing Climate*, ed. H.-O. Pörtner, D.C. Roberts, V. Masson-Delmotte, P. Zhai, M. Tignor, E. Poloczanska, K. Mintenbeck, A. Alegría, et al. In Press, Report.

[CR30] Ives CD, Abson DJ, von Wehrden H, Dorninger C, Klaniecki K, Fischer J (2018). Reconnecting with nature for sustainability. Sustainability Science.

[CR31] Jackowski AV, Grosse J, Nöthig E-M, Engel A (2020). Dynamics of organic matter and bacterial activity in the Fram Strait during summer and autumn. Philosophical Transactions of the Royal Society A.

[CR32] Jarníková T, Dacey J, Lizotte M, Levasseur M, Tortell P (2018). The distribution of methylated sulfur compounds, DMS and DMSP, in Canadian subarctic and Arctic marine waters during summer 2015. Biogeosciences.

[CR33] King GM, Weber CF (2007). Distribution, diversity and ecology of aerobic CO-oxidizing bacteria. Nature Reviews Microbiology.

[CR34] Korhonen H, Carslaw KS, Spracklen DV, Mann GW, Woodhouse MT (2008). Influence of oceanic dimethyl sulfide emissions on cloud condensation nuclei concentrations and seasonality over the remote Southern Hemisphere oceans: A global model study. Journal of Geophysical Research.

[CR35] Krahmann G, Arévalo-Martínez DL, Dale AW, Dengler M, Engel A, Glock N, Grasse P, Hahn J (2021). Climate-biogeochemistry interactions in the tropical ocean: Data collection and legacy. Earth Syst. Sci. Data Discuss..

[CR36] Kwok R (2018). Arctic sea ice thickness, volume, and multiyear ice coverage: losses and coupled variability (1958–2018). Environmental Research Letters.

[CR37] Kwon YS, Kang H-W, Polimene L, Rhee TS (2020). A marine carbon monoxide (CO) model with a new parameterization of microbial oxidation. Ecological Modelling.

[CR38] Lannuzel D, Tedesco L, van Leeuwe M, Campbell K, Flores H, Delille B, Miller L, Stefels J (2020). The future of Arctic sea-ice biogeochemistry and ice-associated ecosystems. Nature Climate Change.

[CR39] Levasseur M (2013). Impact of Arctic meltdown on the microbial cycling of sulphur. Nature Geoscience.

[CR40] Loose B, Miller LA, Elliott S, Papakyriakou T (2011). Sea ice biogeochemistry and material transport across the frozen interface. Oceanography.

[CR41] Malmstrom RR, Straza TR, Cottrell MT, Kirchman DL (2007). Diversity, abundance, and biomass production of bacterial groups in the western Arctic Ocean. Aquatic Microbial Ecology.

[CR42] Meier WN, Hovelsrud GK, Van Oort BE, Key JR, Kovacs KM, Michel C, Haas C, Granskog MA (2014). Arctic sea ice in transformation: A review of recent observed changes and impacts on biology and human activity. Reviews of Geophysics.

[CR43] Meneghello G, Marshall J, MarshallScott MLJ (2018). Observations of seasonal upwelling and Downwelling in the Beaufort sea mediated by sea ice. Journal of Physical Oceanography.

[CR44] Moran MA, Miller WL (2007). Resourceful heterotrophs make the most of light in the coastal ocean. Nature Reviews Microbiology.

[CR45] Motard-Côté J, Levasseur M, Scarratt M, Michaud S, Gratton Y, Rivkin RB, Keats K, Gosselin M (2012). Distribution and metabolism of dimethylsulfoniopropionate (DMSP) and phylogenetic affiliation of DMSP-assimilating bacteria in northern Baffin Bay/Lancaster Sound. Journal of Geophysical Research.

[CR46] Mungall EL, Croft B, Lizotte M, Thomas JL, Murphy JG, Levasseur M, Martin RV, Wentzell JJ (2016). Dimethyl sulfide in the summertime Arctic atmosphere: Measurements and source sensitivity simulations. Atmospheric Chemistry and Physics.

[CR47] Park K-T, Yoon YJ, Lee K, Tunved P, Krejci R, Ström J, Jang E, Kang HJ (2021). Dimethyl sulfide-induced increase in cloud condensation nuclei in the arctic atmosphere. Global Biogeochemical Cycles.

[CR48] Park KT, Lee K, Kim TW, Yoon YJ, Jang EH, Jang S, Lee BY, Hermansen O (2018). Atmospheric DMS in the Arctic Ocean and its relation to phytoplankton biomass. Global Biogeochemical Cycles.

[CR49] Pistone K, Eisenman I, Ramanathan V (2014). Observational determination of albedo decrease caused by vanishing Arctic sea ice. Proceedings of the National Academy of Sciences.

[CR50] Prather MJ (1996). Time scales in atmospheric chemistry: Theory, GWPs for CH4 and CO, and runaway growth. Geophysical Research Letters.

[CR73] Qu B, Gabric AJ, Jackson R (2021). Simulated perturbation in the sea-to-air flux of dimethylsulfide and the impact on polar climate. Journal of Oceanology and Limnology.

[CR51] Semiletov I, Pipko I, Gustafsson Ö, Anderson LG, Sergienko V, Pugach S, Dudarev O, Charkin A (2016). Acidification of East Siberian Arctic Shelf waters through addition of freshwater and terrestrial carbon. Nature Geoscience.

[CR52] Sipler RE, Kellogg CTE, Connelly TL, Roberts QN, Yager PL, Bronk DA (2017). Microbial community response to terrestrially derived dissolved organic matter in the coastal arctic. Frontiers in Microbiology.

[CR53] Song G, Xie H, Aubry C, Zhang Y, Gosselin M, Mundy C, Philippe B, Papakyriakou TN (2011). Spatiotemporal variations of dissolved organic carbon and carbon monoxide in first-year sea ice in the western Canadian Arctic. Journal of Geophysical Research.

[CR54] Song G, Xie H (2017). Spectral efficiencies of carbon monoxide photoproduction from particulate and dissolved organic matter in laboratory cultures of Arctic sea ice algae. Marine Chemistry.

[CR55] Stedmon C, Amon R, Rinehart A, Walker S (2011). The supply and characteristics of colored dissolved organic matter (CDOM) in the Arctic Ocean: Pan Arctic trends and differences. Marine Chemistry.

[CR56] Stefels J (2000). Physiological aspects of the production and conversion of DMSP in marine algae and higher plants. Journal of Sea Research.

[CR57] Stefels J, Steinke M, Turner S, Malin G, Belviso S (2007). Environmental constraints on the production and removal of the climatically active gas dimethylsulphide (DMS) and implications for ecosystem modelling. Biogeochemistry.

[CR58] Sunda W, Kieber D, Kiene R, Huntsman S (2002). An antioxidant function for DMSP and DMS in marine algae. Nature.

[CR59] Taalba A, Xie H, Scarratt MG, Bélanger S, Levasseur M (2013). Photooxidation of dimethylsulfide (DMS) in the Canadian Arctic. Biogeosciences.

[CR60] Terhaar J, Kwiatkowski L, Bopp L (2020). Emergent constraint on Arctic Ocean acidification in the twenty-first century. Nature.

[CR61] Thackeray CW, Hall A (2019). An emergent constraint on future Arctic sea-ice albedo feedback. Nature Climate Change.

[CR62] Tisserand L, Dadaglio L, Intertaglia L, Catala P, Panagiotopoulos C, Obernosterer I, Joux F (2020). Use of organic exudates from two polar diatoms by bacterial isolates from the Arctic Ocean. Philosophical Transactions of the Royal Society A.

[CR63] Todd JD, Kirkwood M, Newton-Payne S, Johnston AWB (2012). DddW, a third DMSP lyase in a model Roseobacter marine bacterium, Ruegeria pomeroyi DSS-3. The ISME Journal.

[CR64] Tran S, Bonsang B, Gros V, Peeken I, Sarda-Esteve R, Bernhardt A, Belviso S (2013). A survey of carbon monoxide and non-methane hydrocarbons in the Arctic Ocean during summer 2010. Biogeosciences.

[CR65] Underwood GJC, Michel C, Meisterhans G, Niemi A, Belzile C, Witt M, Dumbrell AJ, Koch BP (2019). Organic matter from Arctic sea-ice loss alters bacterial community structure and function. Nature Climate Change.

[CR66] Vancoppenolle M, Meiners KM, Michel C, Bopp L, Brabant F, Carnat G, Delille B, Lannuzel D (2013). Role of sea ice in global biogeochemical cycles: Emerging views and challenges. Quaternary Science Reviews.

[CR67] Xie H, Gosselin M (2005). Photoproduction of carbon monoxide in first-year sea ice in Franklin Bay, southeastern Beaufort Sea. Geophysical Research Letters.

[CR68] Xie H, Zafiriou OC, Umile TP, Kieber DJ (2005). Biological consumption of carbon monoxide in Delaware Bay, NW Atlantic and Beaufort Sea. Marine Ecology Progress Series.

[CR69] Xie H, Bélanger S, Demers S, Vincent WF, Papakyriakou TN (2009). Photobiogeochemical cycling of carbon monoxide in the southeastern Beaufort Sea in spring and autumn. Limnology and Oceanography.

[CR70] Zafiriou OC, Xie H, Nelson NB, Najjar RG, Wang W (2008). Diel carbon monoxide cycling in the upper Sargasso Sea near Bermuda at the onset of spring and in midsummer. Limnology and Oceanography.

[CR71] Zeng Y-X, Qiao Z-Y, Yu Y, Li H-R, Luo W (2016). Diversity of bacterial dimethylsulfoniopropionate degradation genes in surface seawater of Arctic Kongsfjorden. Scientific Reports.

[CR72] Zhang Ya, Xie H, Fichot CG, Chen G (2008). Dark production of carbon monoxide (CO) from dissolved organic matter in the St Lawrence estuarine system: Implication for the global coastal and blue water CO budgets. Journal of Geophysical Research.

[CR74] Zheng, B., F. Chevallier, Y. Yin, P. Ciais, A. Fortems-Cheiney, M. N. Deeter, R. J. Parker, Y. Wang, et al. 2019. Global atmospheric carbon monoxide budget 2000–2017 inferred from multi-species atmospheric inversions. *Earth System Science Data* 11: 1411–1436. 10.5194/essd-11-1411-2019.

